# Risk Prediction Models for New Vertebral Fracture After Vertebral Augmentation in Elderly Patients with Osteoporotic Vertebral Compression Fractures: A Systematic Review

**DOI:** 10.3390/healthcare14142162

**Published:** 2026-07-17

**Authors:** Bo He, Wenling Tian, Xiangyue Liu, Xuemei Zheng, Mengjing Chang, Xue Deng, Dongfa Liao, Lin Cui

**Affiliations:** 1 School of Nursing, North Sichuan Medical College, Nanchong 637000, China; hebo@nsmc.edu.cn (B.H.);; 2 Orthopedic Department, Western Theater Command General Hospital, Chengdu 610000, China; 3 School of Nursing, Chengdu Medical College, Chengdu 610000, China; 4 Nursing Department, Western Theater Command General Hospital, Chengdu 610000, China

**Keywords:** osteoporotic vertebral compression fracture, vertebral augmentation, new vertebral fracture, prediction model, systematic review

## Abstract

**Highlights:**

**What are the main findings?**

- Twenty-nine prediction models for new vertebral fracture after vertebral augmentation in elderly patients with osteoporotic vertebral compression fractures were systematically reviewed.
- All included studies were judged to have high overall risk of bias per the PROBAST. Although most models presented acceptable discriminative performance, pervasive methodological flaws severely limit their reliability and generalizability.
- External validation was extremely inadequate across the literature, and deficiencies in statistical analysis represented the most prominent source of bias.

**What are the implications of the main findings?**

- Bone mineral density, bone cement leakage, and anti-osteoporosis treatment were the most frequently identified predictors and may serve as key targets for postoperative risk management.
- Due to the universal high risk of bias, current prediction models are only suitable for research purposes rather than routine clinical application. Future studies must strictly standardize model development, reduce bias, strengthen external validation, and explore machine learning approaches to develop clinically reliable tools.

**Abstract:**

**Objective:** This study aimed to systematically evaluate risk prediction models for new vertebral fracture after vertebral augmentation in elderly patients with osteoporotic vertebral compression fractures (OVCFs) and to summarize their modeling methods, predictors, predictive performance, and methodological quality. **Methods:** This systematic review was prospectively registered in the Open Science Framework (OSF; DOI:10.17605/OSF.IO/S259R). PubMed, Embase, Web of Science, Cochrane Library, CNKI, Wanfang, and VIP were searched from inception to January 2026. Studies reporting model development, internal validation, external validation, or model updating for prediction models of new vertebral fracture after vertebral augmentation in elderly patients with OVCFs were included. Two reviewers independently screened the literature and extracted data. Methodological quality and risk of bias were assessed using the Prediction Model Risk of Bias Assessment Tool (PROBAST). **Results:** A total of 24 studies containing 29 prediction models were included, all of which were conducted in China. The incidence of new vertebral fracture after vertebral augmentation ranged from 10.60% to 38.08%. Logistic regression and nomograms were the most common modeling method and presentation format, respectively. The AUC values of the models ranged from 0.648 to 0.990. Only three studies conducted external validation. Bone mineral density, bone cement leakage, and anti-osteoporosis treatment were the leading predictive factors. PROBAST assessment confirmed that all included studies had a high overall risk of bias, with the statistical analysis domain being the most problematic area. Fourteen studies reported decision curve analysis (DCA), suggesting potential clinical utility within the development datasets. However, no study evaluated real-world clinical impact, and the overall high risk of bias and limited external validation substantially restrict confidence in clinical applicability. **Conclusions:** Although several models demonstrated moderate-to-good discrimination performance, methodological limitations, high overall risk of bias, and insufficient external validation substantially limit confidence in their clinical applicability. These models are not reliable for routine clinical use and should currently be regarded as research tools. Standardized study design, bias control, adequate external validation, and optimized statistical strategies are urgently required to establish credible prediction models for clinical practice.

## 1. Introduction

Osteoporotic vertebral compression fractures (OVCFs) refer to fractures of the thoracic or lumbar vertebrae occurring under minimal or no obvious external trauma and represent one of the most common and severe complications of osteoporosis in older adults [1]. With the acceleration of population aging, osteoporosis has become one of the most prevalent chronic skeletal diseases worldwide, with a global prevalence of approximately 18.3%, and remains a major global public health concern [2]. China has the largest and fastest-growing elderly population worldwide. Studies have shown that approximately 90 million older adults in China suffer from osteoporosis, and osteoporotic vertebral compression fractures have become a major cause of disability and mortality among the elderly, imposing a substantial medical and economic burden on families and society [3].

Surgical intervention is currently an effective treatment option for OVCFs and can facilitate the restoration of spinal structure and stability. Percutaneous vertebral augmentation (PVA), including percutaneous vertebroplasty (PVP) and percutaneous kyphoplasty (PKP), is characterized by short operative time, minimal invasiveness, rapid postoperative recovery, and significant pain relief and has become the preferred surgical treatment for OVCFs [4]. However, clinical studies have reported that the incidence of new vertebral fracture after vertebral augmentation ranges from 20% to 30%, which not only exacerbates pain and functional impairment but also increases the risks of spinal deformity, spinal cord injury, and even mortality, posing a major challenge in postoperative management of elderly patients with OVCFs [5].

Risk prediction models can accurately identify patients at high risk of new vertebral fracture after vertebral augmentation based on multi-dimensional risk factors, thereby providing a basis for individualized prevention and intervention strategies. In recent years, several prediction models have been developed to estimate the risk of new vertebral fracture after PVA in elderly patients with OVCFs. Unlike traditional risk factor studies that primarily identify associations between individual predictors and outcomes, prediction-model studies aim to estimate individualized risk and to support clinical decision-making. Nevertheless, the predictive performance and clinical translation value of these models remain unclear, and no model has been widely accepted for routine clinical practice. Moreover, systematic synthesis and methodological evaluation of prediction models for new vertebral fracture in elderly patients with OVCFs are still lacking. Therefore, this study systematically evaluated prediction models for new vertebral fracture after PVA in elderly patients with OVCFs and analyzed the limitations of current model research, with the aim of providing references for future model development and application, as well as evidence-based clues for clinical identification of high-risk factors. This study was reported in accordance with the Preferred Reporting Items for Systematic Reviews and Meta-Analyses (PRISMA 2020) statement and was registered in the Open Science Framework (OSF) (DOI:10.17605/OSF.IO/S259R). Due to substantial heterogeneity among the included studies, a qualitative synthesis was conducted instead of a meta-analysis.

## 2. Methods

### 2.1. Research Questions

(1) What is the current status of research on prediction models for new vertebral fracture after vertebral augmentation in elderly patients with OVCFs, including modeling methods, predictors, and model presentation formats?
(2) What is the predictive performance, validation status, methodological quality, and clinical applicability of the existing models?
(3) What limitations exist in the current studies, and what implications do they provide for future research?

### 2.2. Literature Search Strategy

**Figure 1:**
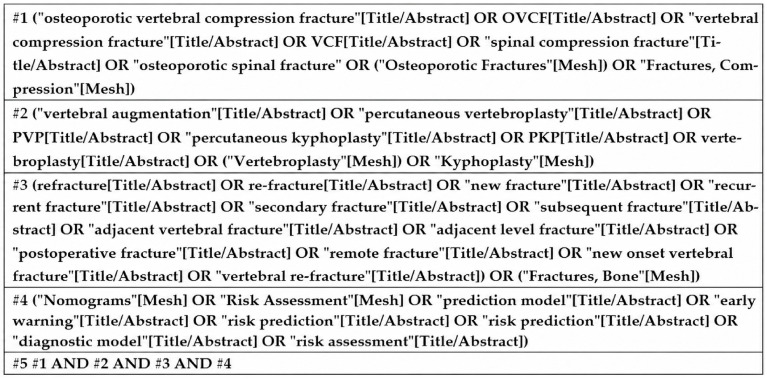
PubMed search strategy.

Search strategies were developed by combining subject headings and free-text terms. A systematic search was conducted in seven Chinese and English databases, including China National Knowledge Infrastructure (CNKI), Wanfang Data, VIP Database, PubMed, Embase, the Cochrane Library, and Web of Science. In addition, the reference lists of the included studies were manually screened to identify potentially relevant publications. The search was updated through February 2026 to capture the most recent eligible studies available at the time the review was conducted. Taking Wanfang Data as an example, the search strategy was formulated as follows: su = (“osteoporotic vertebral compression fracture” OR “osteoporotic fracture”) AND su = (“percutaneous vertebroplasty” OR “vertebral augmentation” OR “percutaneous kyphoplasty”) AND su = (refracture OR “new fracture” OR “secondary fracture” OR “recurrent fracture”) AND su = (prediction OR assessment OR model OR screening OR factor). PubMed was used as an example for the English database search, and the fully reformatted PubMed search strategy is presented in Figure 1. The complete search strategies for all seven databases are provided in Supplementary Material S1.

### 2.3. Eligibility Criteria

The inclusion criteria were as follows:

(1) Study population: Studies were included only if ≥90% of participants were aged ≥60 years and the results for this age subgroup were reported separately. All participants must have met the WHO diagnostic criteria for osteoporosis [6] and the imaging diagnostic criteria for OVCFs [7] and undergone PVA treatment (clearly defined as PVP or PKP). Studies with mixed-age populations that did not provide stratified data, had unclear osteoporosis diagnostic criteria, or had undefined surgical procedures were excluded.
(2) Study content: Studies involving prediction models for postoperative vertebral refracture after PVA, including model development, validation, or updating were included. An eligible prediction model was defined as any multivariable statistical model developed to estimate the individual risk of postoperative refracture, regardless of presentation format (nomogram, risk score, web calculator). Studies using machine learning algorithms were also considered eligible if they generated individualized risk predictions, regardless of whether the term “prediction model” was explicitly used by the original authors. Studies that only performed univariate or multivariate risk factor analysis without constructing a tool for individual risk assessment were excluded.
(3) Study design: Cohort studies or case–control studies reporting the development, validation, or updating of multivariable prediction models for new vertebral fracture after vertebral augmentation.
(4) Outcome definition: For the purpose of this review, the primary outcome was defined as new vertebral fracture after vertebral augmentation, including fractures occurring at adjacent or non-adjacent vertebral levels after the index vertebral augmentation procedure. Adjacent vertebral fracture was defined as a fracture occurring in the vertebral body immediately adjacent to the treated vertebra. Terms used in the original studies, including refracture, recurrent fracture, secondary fracture, and postoperative vertebral fracture, were standardized as new vertebral fracture in this review. We additionally recorded whether each study specifically restricted outcomes to adjacent vertebral fractures.

The exclusion criteria were as follows:

(1) Non-original studies, including reviews, conference abstracts, and basic experimental studies;
(2) Studies with unavailable full texts, duplicate publications, or insufficient data for extraction;
(3) Studies published in languages other than Chinese or English;
(4) Studies that only analyzed risk factors without developing a prediction model.

### 2.4. Study Selection and Data Extraction

Study selection and data extraction were independently performed and cross-checked by two researchers trained in evidence-based medicine. Disagreements were resolved through consultation with a third researcher. Formal inter-reviewer agreement statistics (e.g., Cohen’s kappa) were not calculated. After importing all retrieved records into NoteExpress (Version 4.0, Beijing, China) software and removing duplicates, the two reviewers screened the studies according to the eligibility criteria. Data extraction was conducted based on the CHARMS checklist [8], including the first author, publication year, study design, study population, sample size, modeling methods, predictors, and other relevant information. Regarding the outcome definition, we specifically extracted: (a) whether new vertebral fracture was restricted to adjacent vertebral fractures or included any vertebral level; and (b) the follow-up duration for outcome assessment. This information was used to stratify the qualitative synthesis and to discuss heterogeneity across studies.

### 2.5. Risk of Bias and Applicability Assessment

The Prediction Model Risk of Bias Assessment Tool (PROBAST) was used to evaluate the risk of bias of the included studies [9]. PROBAST evaluates four domains, including participants, predictors, outcomes, and statistical analysis, with each domain rated as having a high, low, or unclear risk of bias. A study was considered to have an overall low risk of bias when all domains were rated as low-risk, whereas the presence of a high risk in any single domain resulted in an overall high risk of bias. If one domain was rated as unclear-risk while the remaining domains were rated as low-risk, the overall risk of bias was judged as unclear. In addition, clinical applicability was assessed across three domains, including participants, predictors, and outcomes. Models were considered to have good applicability when all three domains were rated as low-concern. All included prediction models, including machine learning-based models, were assessed using the same PROBAST framework to ensure consistency across methodological evaluations. Risk-of-bias and applicability assessments were independently conducted by two reviewers, and discrepancies were resolved through discussion with a third reviewer.

## 3. Results

### 3.1. Study Selection Process and Results

**Figure 2:**
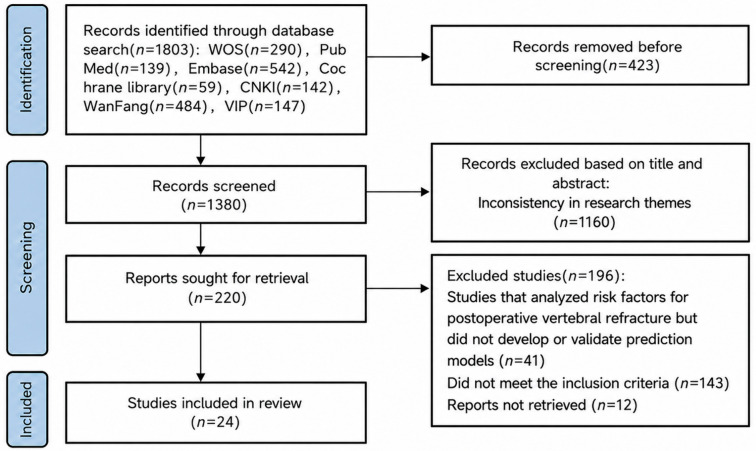
Flow chart of literature screening.

A total of 1803 potentially relevant records were initially identified. After sequential screening, 24 studies were ultimately included in this review. The detailed study selection process is presented in the PRISMA flow diagram (Figure 2).

### 3.2. Basic Characteristics of the Included Studies

A total of 24 studies published between 2021 and 2026 were ultimately included in this review, all conducted in China. We systematically summarized the basic characteristics according to four dimensions: study design, outcome type, modeling method, and validation status. A concise core summary is presented in Table 1, and full detailed information is provided in Supplementary Table S1.

Study design: All included studies adopted a retrospective cohort design. Apart from one multi-center study by Bian et al. [10], all were single-center studies. The sample sizes ranged from 150 to 562 participants, with postoperative follow-up durations ranging from 6 to 36 months. Across all studies, the number of new vertebral fracture events ranged from 31 to 188 cases, and the overall incidence of new vertebral fracture after vertebral augmentation ranged from 10.60% to 38.08%.

Outcome type: According to the anatomical definition of new vertebral fracture, the included studies were divided into two distinct groups: six studies explicitly restricted the outcome to adjacent vertebral fractures only [11,12,13,14,15,16], while the remaining 18 studies did not specify anatomical location, thus including both adjacent and non-adjacent vertebral fractures. This difference in outcome definition was identified as a key source of clinical and methodological heterogeneity across studies.

Modeling methods and presentation formats: Four types of statistical approaches were used to develop the 29 prediction models: logistic regression [10,11,12,13,17,18,19,20,21,22,23,24,25,26,27,28,29], LASSO regression combined with logistic regression [14,15,30,31,32], Cox proportional hazards regression [16], and machine learning [33]. Nomograms were the most common presentation format, followed by risk-scoring formulas. Two models [27,29] additionally developed web-based calculators. Traditional logistic regression was the most frequently used modeling approach and was favored because of its simplicity and interpretability. LASSO-based models incorporated penalized regression techniques for predictor selection and may reduce the risk of overfitting compared with conventional regression approaches. Only one study [33] applied machine learning algorithms. Although machine learning methods demonstrated promising discriminative performance, their interpretability and external validity remain insufficiently evaluated. Therefore, no clear superiority of machine learning models over regression-based models could be established based on the currently available evidence.

Validation status: All included studies were model development studies; no model-updating studies or external validation-only studies were identified. Five studies did not perform any validation [18,21,22,25,26], 16 studies conducted internal validation only [11,12,13,14,15,17,19,23,24,27,28,29,30,31,32,33], and only 3 studies performed both internal and independent external validation [10,16,20].

Reporting guideline adherence was rarely reported. Among the 24 included studies, one study [27] reported adherence to the STROCSS guideline and one study [15] reported adherence to the TREND statement, whereas none explicitly reported compliance with the TRIPOD guideline or other prediction-model-specific reporting standards.

### 3.3. Predictors Included in the Models

The included prediction models incorporated 2 to 14 predictors each (Table 2). All predictors were categorized into five predefined domains: demographic characteristics, disease-related factors, surgery-related factors, treatment-related factors, and other factors. Detailed frequency information for all predictors is presented in Table 3, and predictors reported only once are listed in Supplementary Table S2.

Consistent with clinical expectations, the predictor profiles differed significantly between the two outcome groups, reflecting the distinct pathophysiological mechanisms underlying different types of new vertebral fracture. Models for adjacent vertebral fracture (six studies): These models predominantly emphasized surgery-related factors, which were more strongly associated with procedure-specific biomechanical changes. These models most frequently included bone cement leakage, bone cement injection volume, and bone cement distribution as core predictors. Models for any new vertebral fracture (18 studies): These models placed greater emphasis on systemic and disease-related factors, which reflect the underlying osteoporosis severity and overall health status. These models most frequently included bone mineral density, anti-osteoporosis treatment, and age as core predictors.

Across all 29 prediction models, the five most frequently reported predictors were bone mineral density, bone cement leakage, anti-osteoporosis treatment, bone cement volume, and age.

### 3.4. Predictive Performance of the Models

We systematically evaluated predictive performance across three key dimensions: discrimination performance (stratified by validation type), calibration assessment, and clinical utility metrics. The model-level performance data are shown in Table 2. The detailed predictive performance data of each model are summarized in Supplementary Table S3.

#### 3.4.1. Discrimination Performance

Discrimination ability was primarily assessed using the area under the receiver operating characteristic curve (AUC).

Apparent (training-set) performance was reported in 22 studies [10,11,12,14,15,16,17,18,19,20,21,22,24,25,26,27,28,29,30,31,32,33], with AUC values ranging from 0.648 to 0.990. Twenty studies reported an AUC > 0.700, while only two studies [20,26] reported an AUC < 0.700. Important consideration: These training-set AUC values should be interpreted very cautiously, as 13 studies [11,12,13,15,16,17,19,20,21,23,24,25,32] had fewer than 10 events per variable (EPV), significantly increasing the risk of severe overfitting.

Internal validation was conducted in 16 studies [11,12,13,14,15,17,19,23,24,27,28,29,30,31,32,33], with AUC values ranging from 0.750 to 0.976. Internal validation assesses model stability within the original dataset but cannot confirm predictive accuracy in new, unseen populations.

External validation was performed in only three studies [10,16,20], with AUC values ranging from 0.648 to 0.870. External validation is the gold standard for assessing model generalizability, and these three models represent the most mature tools in the field.

#### 3.4.2. Calibration Assessment

Calibration, which measures the agreement between predicted and observed risks, was assessed using two methods: visual calibration curves [10,11,12,13,14,15,16,17,19,20,23,24,25,26,27,28,29,30,31,32,33] and the Hosmer–Lemeshow (H-L) test [14,16,18,21,22,23,25,26,28,31]. The H-L test results suggested acceptable agreement in the development or internal validation samples, but it should be noted that the H-L test has low statistical power for detecting poor calibration in small samples and should not be considered sufficient evidence on its own.

#### 3.4.3. Clinical Utility Metrics

Six studies [11,14,18,21,22,33] reported model sensitivity (range 0.768–0.940) and specificity (range 0.583–0.948), indicating moderate predictive accuracy. In addition, 14 studies [10,12,14,15,16,19,23,27,28,29,30,31,32,33] assessed potential clinical utility using decision curve analysis (DCA), generally demonstrating a net benefit across selected threshold probabilities within the study populations. However, no study evaluated model impact in routine clinical practice through prospective implementation or impact analysis.

### 3.5. Risk-of-Bias and Applicability Assessment of the Models

Based on the PROBAST, we assessed the risk of bias and clinical applicability of all 24 included studies across four core domains: participants, predictors, outcomes, and statistical analysis. The assessment results for individual studies are listed in Table 4, and the quantitative distribution of bias across each domain is summarized in Table 5.

## 4. Discussion

### 4.1. Current Status and Clinical Implications of Prediction Models for Postoperative Refracture After PVA in Elderly Patients with OVCFs

The increasing number of prediction-model studies published in recent years reflects growing clinical interest in postoperative refracture risk stratification among elderly patients with OVCFs.

Studies that restricted new vertebral fracture outcomes to adjacent vertebral fractures tended to emphasize surgery-related predictors, such as bone cement leakage and cement distribution, whereas studies without anatomical restrictions placed greater emphasis on patients’ overall systemic conditions. The core predictors frequently reported in existing models mainly included bone mineral density, bone cement leakage, and anti-osteoporosis treatment. These findings from predictor analysis provide candidate directions for clinical prevention strategies, which may focus on three key aspects: preoperative bone mass assessment, standardized intraoperative bone cement procedures, and long-term standardized anti-osteoporosis therapy following surgery.

Notably, several predictors included in the models, such as adherence to anti-osteoporosis treatment, postoperative functional exercise, and medication management, are modifiable nursing-related factors. This finding suggests that future well-validated prediction models incorporating these factors may not only facilitate the identification of high-risk populations but also provide targeted guidance for individualized nursing interventions, thereby contributing to continuity of care and hierarchical management in elderly patients with OVCF after surgery. In addition, for patients at high risk of adjacent vertebral fractures, healthcare professionals should optimize surgical techniques, such as controlling bone cement volume and minimizing cement leakage, while simultaneously strengthening comprehensive systemic management to reduce the incidence of new vertebral fracture after vertebral augmentation. Importantly, the findings of this review should not be interpreted as supporting the routine clinical use of any existing prediction model. Additional validation and implementation studies are still required before these models can be routinely applied in clinical practice.

### 4.2. Predictive Performance and Methodological Limitations of Existing Models

The findings of this review indicate that the study populations, predictors, and outcome indicators of the included models are consistent with real clinical scenarios, so the models possess theoretical clinical potential. Notably, PROBAST evaluation indicated that all included studies presented high overall risk of bias, and methodological defects are the biggest obstacle to the clinical translation of these prediction models. Among all domains, flaws in the statistical analysis domain are most prominent and deserve special attention in prediction-model research.

These limitations were mainly reflected in the following four aspects. First, the study designs had inherent limitations and insufficient generalizability. All included studies were retrospective cohort studies, and only one study [10] was conducted across multiple centers. Single-center retrospective designs are prone to selection bias and information bias, thereby limiting the external applicability of the findings. In addition, the follow-up duration varied substantially across studies (6–36 months), further increasing interstudy heterogeneity. Second, the statistical methods used for model development were often suboptimal, resulting in a high risk of overfitting. Notably, the high AUC values reported in several studies should not be directly interpreted as evidence of clinical applicability. In prediction-model research, models developed using small sample sizes, limited outcome events, or excessive candidate predictors may exhibit overly optimistic apparent discrimination. In this review, 13 studies [11,12,13,15,16,17,19,20,21,23,24,25,32] had fewer than 10 events per variable (EPV), which is substantially below the recommended standard for prediction-model development [34]. For example, in the study by Wang Xulong et al. [25], only 32 new vertebral fracture events were included with five independent variables entered into the model, yielding an EPV of 6.4. This insufficient EPV may result in severe model overfitting, leading to inflated apparent performance and markedly reduced predictive accuracy in real-world applications. Moreover, most studies used univariate analysis for variable selection, which may lead to omission of clinically important predictors. Only five studies [14,15,30,31,32] applied least absolute shrinkage and selection operator (LASSO) regression for predictor selection, demonstrating greater methodological rigor. Regarding missing data, only two studies [27,33] used multiple imputation methods, whereas the remaining studies did not clearly report how missing values were handled, potentially compromising model stability. Third, model validation was insufficient, and the generalizability of the models remains unclear. Five studies [18,21,22,25,26] did not perform standardized internal or external validation, leaving the applicability of the developed models uncertain. Although 16 studies [11,12,13,14,15,17,19,23,24,27,28,29,30,31,32,33] conducted internal validation, such validation can only assess model stability within the original dataset and cannot confirm predictive accuracy in external populations, which represents a major obstacle to clinical implementation. Only three studies [10,16,20] performed independent external validation, suggesting relatively better model generalizability. Fourth, inadequate bias control reduced the reliability of the study findings. None of the included studies reported blinding of predictor assessors or outcome evaluators. In retrospective studies, prior knowledge of patient outcomes may introduce assessment bias. Three studies [12,16,21] transformed continuous variables, such as age and bone cement volume, into categorical variables, which may reduce model predictive performance. Future studies should further standardize the development and reporting of prediction models by adhering to established methodological and reporting guidance, including CHARMS [8], PROBAST [9], TRIPOD [35], and TRIPOD-AI [36] recommendations. Although 14 studies [10,12,14,15,16,19,23,27,28,29,30,31,32,33] reported favorable DCA results, DCA only evaluates potential net benefit under hypothetical decision thresholds and does not constitute evidence of real-world clinical effectiveness. Therefore, the reported DCA findings should be interpreted cautiously, particularly given the universal high risk of bias and limited external validation.

### 4.3. Knowledge Gaps and Future Research Directions

This review systematically summarized the major limitations of existing studies and identified several important knowledge gaps in this field, providing directions for future research. First, methodological standards for model development require further standardization. Most existing studies were retrospective and single-center in design, with a lack of high-quality multi-center prospective studies. Future studies should prioritize multi-center prospective designs with adequate sample sizes and outcome events to improve robustness and generalizability. Second, external validation and clinical implementation studies remain insufficient. Future studies should prioritize validation of promising existing models across different populations and healthcare settings to improve generalizability and support clinical translation. In addition, future research should move beyond static baseline prediction models and develop dynamic longitudinal prediction models that allow time-updated estimation of postoperative fracture risk during follow-up. Third, the dimensions of predictors and population specificity require further expansion. Existing models primarily focus on clinical and surgery-related variables, while insufficient attention has been paid to factors such as sarcopenia [37], frailty [38], and social support [39] in elderly patients. These factors are important determinants of adverse postoperative outcomes in OVCFs and represent potentially modifiable intervention targets. In addition, multimorbidity is highly prevalent among elderly patients with OVCFs [40], yet current models rarely address populations with multiple chronic conditions. Only one study [33] incorporated predictors related to multimorbidity. This study focused on elderly patients with underlying chronic diseases and used machine learning techniques to identify overlooked but influential predictors, such as scoliosis, psychiatric disorders, and chronic kidney disease. These factors, together with multimorbidity, may substantially increase the risk of new vertebral fracture after vertebral augmentation. Future studies should broaden the scope of predictors and develop targeted prediction models for specific subgroups, such as patients with multimorbidity or frailty, to enhance clinical applicability. Fourth, most existing models rely on traditional logistic regression, which has limited the ability to address the nonlinear relationships and multicollinearity commonly present in clinical data [41]. Machine learning algorithms have demonstrated significant advantages in handling high-dimensional data and identifying complex interactions among risk factors [42]. Future studies may further explore the application of machine learning approaches in this field while ensuring standardized model development and validation procedures to avoid overfitting and improve predictive accuracy. Future machine learning prediction models should emphasize explainability and interpretability. Emerging explainable artificial intelligence (XAI) approaches, such as SHAP (Shapley Additive Explanations) values and LIME (Local Interpretable Model-Agnostic Explanations), may improve transparency, facilitate interpretation of model predictions, and enhance clinician acceptance while maintaining predictive performance. Fifth, there is a lack of standardized definitions for refracture outcomes. Among the included studies, only six restricted new vertebral fracture outcomes according to anatomical location, whereas the remaining studies did not specify fracture sites. Such heterogeneity in outcome definitions may hinder objective comparisons of predictor weights and model performance across studies and may further impede clinical implementation. Therefore, establishing standardized criteria for refracture outcomes is urgently needed to improve study homogeneity and model comparability. Sixth, reporting quality remains suboptimal. Only two studies [15,27] explicitly reported adherence to established reporting guidelines (STROCSS or TREND) and none reported compliance with TRIPOD, which is specifically recommended for prediction-model studies. Future research should follow TRIPOD and TRIPOD-AI reporting standards to improve transparency, reproducibility, and methodological rigor.

### 4.4. Limitations of This Review

First, all included studies were conducted in China. Differences in patient characteristics, osteoporosis management strategies, healthcare systems, surgical practices, and follow-up protocols across countries may limit the applicability of these models to other populations. Therefore, the generalizability of the existing models remains uncertain until they are externally validated in diverse international settings. Second, because of substantial clinical and methodological heterogeneity among the included studies, a meta-analysis was not performed, which may limit the strength of the conclusions drawn from this systematic review. Clinical heterogeneity existed in patient characteristics, sample sizes, and follow-up durations. Methodological heterogeneity arose from differences in predictor selection strategies, modeling approaches, validation procedures, and performance assessment methods. These differences precluded meaningful quantitative pooling of model performance estimates. Third, most included studies were retrospective cohort studies with considerable heterogeneity, which may have constrained the ability to comprehensively and systematically evaluate the accuracy of the developed prediction models. Fourth, the heterogeneity in outcome definitions across studies (adjacent vertebral fracture vs. any-site vertebral fracture) further limits the comparability of predictor weights and model performance. Adjacent vertebral fracture may depend more strongly on procedure-related factors, such as cement leakage, cement distribution, and cement volume, whereas any-site new vertebral fracture may depend more on systemic osteoporosis, bone mineral density, comorbidities, and anti-osteoporosis treatment. This distinction hinders objective comparison of predictor importance across models and may impede clinical implementation. Therefore, our qualitative synthesis should be interpreted with caution, and future studies should adopt standardized outcome definitions. Fifth, all included studies were assessed as having high overall risk of bias via PROBAST. The methodological defects of primary studies may also affect the robustness of the conclusions of this systematic review. Sixth, only studies published in English or Chinese were included, which may have introduced language bias. Furthermore, publication bias cannot be excluded because unpublished studies, conference abstracts, and other gray literature were not systematically searched. Therefore, the available evidence may overrepresent studies reporting favorable model performance.

## 5. Conclusions

This systematic review evaluated 29 prediction models for new vertebral fracture after vertebral augmentation in elderly OVCF patients from 24 eligible studies. Bone mineral density, bone cement leakage, and anti-osteoporosis treatment were the most frequently identified predictors across included models. Methodologically, all included studies had a high overall risk of bias per PROBAST assessment. Critical limitations include extremely insufficient external validation, inconsistent definitions of new vertebral fracture outcomes, and widespread suboptimal statistical practices (such as inadequate event numbers, unjustified categorization of continuous predictors, non-transparent missing-data handling, and incomplete reporting of calibration and clinical utility). Taken together, all currently available models remain at a preliminary research stage. Existing evidence is insufficient to support their routine clinical implementation for risk stratification, and they should only be applied for academic research purposes at this stage. For future studies, we recommend adopting prospective multi-center designs with adequate sample sizes and outcome events, avoiding arbitrary categorization of continuous predictors, handling missing data transparently, routinely reporting calibration and clinical utility, and testing real-world model impact prior to clinical implementation. Standardized outcome definitions are also urgently needed to improve comparability across studies. High-quality models developed under these standards may ultimately support individualized postoperative risk management for elderly OVCF patients.

## Figures and Tables

**Table 1:** Basic characteristics of the included studies.

Characteristic	Summary
Included studies	24
Models	29
Sample size	150–562
Follow-up	6–36 months
Modeling methods	LR, LASSO-LR, Cox, ML
Validation	Internal/External

**Table 2:** Construction and validation of included prediction models.

Included Study	Validation Method	Predictors	Model Performance Evaluation
Zhou XL et al. [11]	Internal validation	Bone cement leakage; BMD; Anti-osteoporosis treatment status; Bone cement injection volume; Vertebral height recovery rate; Age; Bone cement distribution	AUC = 0.908
Shen Y et al. [16]	Internal validation, External validation	Bone cement leakage; BMD; Bone cement injection volume; Vertebral height recovery rate; Intravertebral cleft sign	1-year postoperative AUC = 0.820 ^b^, 2-year AUC = 0.772 ^b^; 1-year postoperative AUC = 0.778 ^c^, 2-year AUC = 0.721 ^c^
Li X et al. [17]	Internal validation	Bone cement injection volume; Age; Sex; VAS pain score	AUC = 0.744 ^a^; AUC = 0.809 ^b^
Huang H et al. [18]	Not reported	Bone cement leakage; BMD; Number of fractured vertebrae; Bisphosphonate use	AUC = 0.921
Tan HT et al. [19]	Internal validation	BMD; Anti-osteoporosis treatment status; Glucocorticoid use; BMI; Number of fractured vertebrae	AUC = 0.909
Li QJ et al. [12]	Internal validation	Bone cement leakage; Anti-osteoporosis treatment status; Bone cement injection volume; Bone cement distribution	AUC = 0.881
Li WL et al. [20]	Internal validation, External validation	BMD; Anti-osteoporosis treatment status; Glucocorticoid use; BMI; Number of fractured vertebrae	AUC = 0.796 ^b^; AUC = 0.648 ^c^
Li KP et al. [30]	Internal validation	Anti-osteoporosis treatment status; Sex; Heart disease; Poor self-care ability; Gastrointestinal disease	AUC = 0.750
Zhang N et al. [21]	Not reported	BMD; Anti-osteoporosis treatment status; Age; Sex; Decreased serum 25-hydroxyvitamin D3; Number of fractured vertebrae; Regular postoperative exercise	AUC = 0.869
He Y et al. [22]	Not reported	Bone cement leakage; BMD; Glucocorticoid use; Sagittal spinal imbalance; Elevated serum β-crosslaps	AUC = 0.894
Sun L et al. [23]	Internal validation	Bone cement leakage; BMD; Bone cement injection volume; Change in kyphotic angle; Number of fractured vertebrae; Vertebral height difference before and after PKP	C-index = 0.818
Huang D et al. [24]	Internal validation	Bone cement leakage; BMD; Age; Paraspinal muscle mass	AUC = 0.976
Wang XL et al. [25]	Not reported	Bone cement leakage; BMD; Bone cement injection volume; Age; Sagittal spinal imbalance	AUC = 0.885
Zhou QF et al. [13]	Internal validation	BMD; Anti-osteoporosis treatment status; Bone cement injection volume; Vertebral height recovery rate; Bone cement distribution; Initial fracture site	C-index = 0.952
Gai JY et al. [26]	Not reported	BMD; Anti-osteoporosis treatment status; Glucocorticoid use; Regular postoperative exercise	AUC = 0.670
Ma YM et al. [27]	Internal validation	Bone cement leakage; BMD; History of previous fracture; Cerebrovascular disease; Interval from fracture to hospitalization/surgery	AUC = 0.927 ^a^; AUC = 0.807 ^b^
YANG et al. [28]	Internal validation	BMD; Paraspinal muscle mass	AUC = 0.861 ^a^; AUC = 0.796 ^b^
HAIBIER et al. [14]	Internal validation	Bone cement leakage; BMD; Bone cement injection volume; Intravertebral cleft sign; Preoperative Cobb angle; Interval from fracture to hospitalization/surgery	AUC = 0.839 ^a^; AUC = 0.846 ^b^
ZHANG et al. [31]	Internal validation	Vertebral height recovery rate; Preoperative anterior vertebral height	AUC = 0.833 ^a^; AUC = 0.771 ^b^
BAO et al. [33]	Internal validation	History of scoliosis; Diabetes mellitus; Chronic kidney disease; Mental disorder; History of coronary stent implantation; Number of fractured vertebrae; Gout; Hypertension; Heart disease; Trauma history; History of alcohol consumption; Tumor; COPD; Osteoarthritis	RF AUC = 0.990 ^a^, RF AUC = 0.880 ^b^, LR AUC = 0.870 ^b^, XGBoost AUC = 0.870 ^b^, GBM AUC = 0.880 ^b^, MLP AUC = 0.880 ^b^, SVM AUC = 0.860 ^b^
MA et al. [29]	Internal validation	Bone cement leakage; Sex; History of previous fracture; Cerebrovascular disease	AUC = 0.795 ^a^; AUC = 0.861 ^b^
ZHANG et al. [32]	Internal validation	Bone cement leakage; BMD; Anti-osteoporosis treatment status; Vertebral height recovery rate; Age; Bone cement distribution; BMI; Preoperative anterior vertebral height	AUC = 0.881 ^a^; AUC = 0.929 ^b^
MAO et al. [15]	Internal validation	Bone cement leakage; BMD; Bone cement injection volume; Age; Sex; Diabetes mellitus; Fracture at thoracolumbar junction; Number of fractured vertebrae	AUC = 0.886 ^a^; AUC = 0.833 ^b^
BIAN et al. [10]	Internal validation, External validation	Bone cement leakage; BMD; Age; Fracture at thoracolumbar junction	AUC = 0.850 ^a^; AUC = 0.880 ^b^; AUC = 0.870 ^c^

Notes: AUC = Area under the curve; C-index = concordance index; RF = random forest; LR = logistic regression; XGBoost = extreme gradient boosting; GBM = gradient boosting machine; MLP = multilayer perceptron; SVM = support vector machine; PKP = percutaneous kyphoplasty; VAS = visual analog scale; ^a^ = training set; ^b^ = test set; ^c^ = external validation set; BMD = bone mineral density.

**Table 3:** Classification of predictive factors.

Predictor	Frequency (*n*)	Predictor	Frequency (*n*)
Demographic characteristics		Surgery-related factors	
Age	8 [10,11,15,17,21,24,25,32]	Bone cement leakage	14 [10,11,12,14,15,16,18,22,23,24,25,27,29,32]
Sex	5 [15,17,21,29,30]	Bone cement injection volume	9 [11,12,13,14,15,16,17,23,25]
Body mass index	3 [19,20,32]	Vertebral height recovery rate	5 [11,13,16,31,32]
Disease-related factors		Bone cement distribution	4 [11,12,13,32]
Bone mineral density	18 [10,11,13,14,15,16,18,19,20,21,22,23,24,25,26,27,28,32]	Sagittal spinal imbalance	2 [22,25]
Number of fractured vertebrae	7 [15,18,19,20,21,23,33]	Preoperative anterior vertebral height	2 [31,32]
Cerebrovascular disease	2 [27,29]	Interval from fracture to hospitalization/surgery	2 [14,27]
History of previous fracture	2 [27,29]	Treatment-related factors	
Intravertebral cleft sign	2 [14,16]	Anti-osteoporosis treatment status	9 [11,12,13,19,20,21,26,30,32]
Fracture at thoracolumbar junction	2 [10,15]	Glucocorticoid use	4 [19,20,22,26]
Diabetes mellitus	2 [15,33]	Regular postoperative exercise	2 [21,26]
Heart disease	2 [30,33]	Other factors	
		Paraspinal muscle mass	2 [24,28]

**Table 4:** Risk-of-bias and applicability assessment of included studies.

Included Study	Risk of Bias				Applicability			Overall	
	Participants	Predictors	Outcome	Analysis	Participants	Predictors	Outcome	Risk of Bias	Applicability
Zhou XL et al. [11]	H	L	L	H	L	L	L	H	L
Shen Y et al. [16]	H	L	L	H	L	L	L	H	L
Li X et al. [17]	H	L	L	H	L	L	L	H	L
Huang H et al. [18]	H	L	H	L	L	L	L	H	L
Tan HT et al. [19]	H	L	L	H	L	L	L	H	L
Li QJ et al. [12]	H	L	L	H	L	L	L	H	L
Li WL et al. [20]	H	L	L	H	L	L	L	H	L
Li KP et al. [30]	H	L	L	L	L	L	L	H	L
Zhang N et al. [21]	H	L	L	H	L	U	L	H	U
He Y et al. [22]	H	L	L	L	L	L	L	H	L
Sun L et al. [23]	H	L	L	H	L	L	L	H	L
Huang D et al. [24]	H	L	L	H	L	L	L	H	L
Wang XL et al. [25]	H	L	L	H	L	L	L	H	L
Zhou QF et al. [13]	H	L	L	H	L	L	L	H	L
Gai JY et al. [26]	H	U	L	L	L	U	L	H	U
Ma YM et al. [27]	H	L	U	L	L	L	L	H	L
YANG et al. [28]	H	L	L	L	L	L	L	H	L
HAIBIER et al. [14]	H	L	L	L	L	L	L	H	L
ZHANG et al. [31]	H	L	L	L	L	L	L	H	L
BAO et al. [33]	H	U	L	L	L	L	L	H	L
MA et al. [29]	H	L	L	L	L	L	L	H	L
ZHANG et al. [32]	H	L	L	H	L	L	L	H	L
MAO et al. [15]	H	L	L	H	L	L	L	H	L
BIAN et al. [10]	H	L	L	L	L	L	L	H	L

Note: L = Low risk of bias/low concern for applicability; H = high risk of bias/high concern for applicability; U = unclear risk of bias/unclear concern for applicability.

**Table 5:** PROBAST risk-of-bias distribution across four domains.

PROBAST Domain	Low Risk (*n*, %)	High Risk (*n*, %)	Unclear Risk (*n*, %)
Participants	0 (0%)	24 (100%)	0 (0%)
Predictors	22 (91.7%)	0 (0%)	2 (8.3%)
Outcomes	22 (91.7%)	1 (4.2%)	1 (4.2%)
Analysis	11 (45.8%)	13 (54.2%)	0 (0%)

Note: All 24 included studies were rated as having high overall risk of bias. The statistical analysis domain showed the most severe methodological defects.

## Data Availability

No new data were created or analyzed in this study. Data sharing is not applicable to this article.

## Supplementary Materials

The following supporting information can be downloaded at https://www.mdpi.com/article/10.3390/healthcare14142162/s1, Supplementary Material S1: Full search strategies; Supplementary Table S1: Detailed characteristics of the included studies and prediction models; Supplementary Table S2: Frequency of predictors identified in the included studies, including predictors reported only once; Supplementary Table S3: Detailed information on model development and validation of the included prediction models; PRISMA 2020 checklist.
