# Supplementary material for: Risk Prediction Models for New Vertebral Fracture After Vertebral Augmentation in Elderly Patients with Osteoporotic Vertebral Compression Fractures: A Systematic Review
Source: Healthcare (Basel). 2026 Jul 17;14(14):2162. doi: 10.3390/healthcare14142162 (PMC13411060; doi:10.3390/healthcare14142162)
Supplement: Supplementary file 1 [file healthcare-14-02162-s001.zip › Supplementary Material S1.pdf]

Full Search Strategies

Search period: From inception to February 2026.

Language restriction: Chinese and English.

Databases searched: PubMed, Embase, Web of Science, Cochrane Library, CNKI, Wanfang Data, VIP,

1. PubMed

#1 ("osteoporotic vertebral compression fracture"[Title/Abstract] OR OVCF[Title/Abstract] OR "vertebral compression fracture"[Title/Abstract] OR VCF[Title/Abstract] OR "spinal compression fracture"[Title/Abstract] OR "osteoporotic spinal fracture" OR ("Osteoporotic Fractures"[Mesh]) OR "Fractures, Compression"[Mesh])
#2 ("vertebral augmentation"[Title/Abstract] OR "percutaneous vertebroplasty"[Title/Abstract] OR PVP[Title/Abstract] OR "percutaneous kyphoplasty"[Title/Abstract] OR PKP[Title/Abstract] OR vertebroplasty[Title/Abstract] OR ("Vertebroplasty"[Mesh]) OR "Kyphoplasty"[Mesh])
#3 (refracture[Title/Abstract] OR re-fracture[Title/Abstract] OR "new fracture"[Title/Abstract] OR "recurrent fracture"[Title/Abstract] OR "secondary fracture"[Title/Abstract] OR "subsequent fracture"[Title/Abstract] OR "adjacent vertebral fracture"[Title/Abstract] OR "adjacent level fracture"[Title/Abstract] OR "postoperative fracture"[Title/Abstract] OR "remote fracture"[Title/Abstract] OR "new onset vertebral fracture"[Title/Abstract] OR "vertebral re-fracture"[Title/Abstract]) OR ("Fractures, Bone"[Mesh])
#4("Nomograms"[Mesh] OR "Risk Assessment"[Mesh]) OR "prediction model"[Title/Abstract] OR "early warning"[Title/Abstract] OR "risk prediction"[Title/Abstract] OR "risk prediction"[Title/Abstract] OR "diagnostic model"[Title/Abstract] OR "risk assessment"[Title/Abstract])
#1 AND #2 AND #3 AND #4

2. Embase

('osteoporotic vertebral compression fracture'/exp OR 'osteoporotic vertebral compression fracture' OR ovcf OR 'vertebral compression fracture'/exp OR 'vertebral compression fracture' OR 'vcf'/exp OR vcf OR 'spinal compression fracture' OR 'osteoporotic spinal fracture' OR 'osteoporotic fractures'/exp OR 'osteoporotic fractures' OR 'fractures, compression'/exp OR 'fractures, compression') AND ('vertebral augmentation'/exp OR 'vertebral augmentation' OR 'percutaneous vertebroplasty'/exp OR 'percutaneous vertebroplasty' OR 'pvp'/exp OR pvp OR 'percutaneous kyphoplasty'/exp OR 'percutaneous kyphoplasty' OR pkp OR 'vertebroplasty'/exp OR vertebroplasty OR

'vertebroplasty'/exp OR 'vertebroplasty' OR 'kyphoplasty'/exp OR 'kyphoplasty') AND ('refracture'/exp OR refracture OR 're fracture' OR 'new fracture' OR 'recurrent fracture'/exp OR 'recurrent fracture' OR 'secondary fracture'/exp OR 'secondary fracture' OR 'subsequent fracture' OR 'adjacent vertebral fracture'/exp OR 'adjacent vertebral fracture' OR 'adjacent level fracture' OR 'postoperative fracture'/exp OR 'postoperative fracture' OR 'remote fracture' OR 'new onset vertebral fracture' OR 'vertebral re-fracture' OR 'fractures, bone'/exp OR 'fractures, bone') AND ('nomograms'/exp OR 'nomograms' OR 'prediction model'/exp OR 'prediction model' OR 'early warning' OR 'risk prediction'/exp OR 'risk prediction' OR 'diagnostic model'/exp OR 'diagnostic model' OR 'risk assessment'/exp OR 'risk assessment')

3. Web of Science

#1 vertebral augmentation OR percutaneous vertebroplasty OR PVP OR percutaneous kyphoplasty OR PKP OR vertebroplasty OR Vertebroplasty OR Kyphoplasty (Topic)
#2 osteoporotic vertebral compression fracture OR OVCF OR vertebral compression fracture OR VCF OR spinal compression fracture OR osteoporotic spinal fracture OR Osteoporotic Fractures OR Fractures, Compression (Topic)
#3 vertebral augmentation OR percutaneous vertebroplasty OR PVP OR percutaneous kyphoplasty OR PKP OR vertebroplasty OR Vertebroplasty OR Kyphoplasty (Topic)
#4 Nomograms OR Risk Assessment OR prediction model OR early warning OR risk prediction OR diagnostic model OR risk assessment (Topic)
#1 AND #2 AND #3 AND #4

4. Cochrane Library

(Nomograms OR Risk Assessment OR prediction model OR early warning OR risk prediction OR diagnostic model OR risk assessment):ti,ab,kw AND (refracture OR re-fracture OR new fracture OR recurrent fracture OR secondary fracture OR subsequent fracture OR adjacent vertebral fracture OR adjacent level fracture OR postoperative fracture OR remote fracture OR new onset vertebral fracture OR vertebral re-fracture OR Fractures, Bone):ti,ab,kw AND (vertebral augmentation OR percutaneous vertebroplasty OR PVP OR percutaneous kyphoplasty OR PKP OR vertebroplasty OR Vertebroplasty OR Kyphoplasty):ti,ab,kw AND (osteoporotic vertebral compression fracture OR OVCF OR vertebral compression fracture OR VCF OR spinal compression fracture OR osteoporotic spinal fracture OR Osteoporotic Fractures OR Fractures, Compression):ti,ab,kw" (Word variations have been searched)

5.CNKI

#1 主题：骨质疏松性椎体压缩性骨折 + 骨质疏松性骨折
#2 主题：经皮椎体成形术 + 椎体强化术 + 经皮椎体后凸成形术
#3 主题：再骨折 + 新发骨折 + 二次骨折 + 复发骨折
#4 主题：预测 + 评估 + 模型 + 筛查 + 因素
#5: #1 and #2 and #3 and #4

6.Wanfang Data

#1 主题：骨质疏松性椎体压缩性骨折 or 骨质疏松性骨折
#2 主题：经皮椎体成形术 or 椎体强化术 or 经皮椎体后凸成形术
#3 主题：再骨折 or 新发骨折 or 二次骨折 or 复发骨折
#4 主题：预测 or 评估 or 模型 or 筛查 or 因素
#5: #1 and #2 and #3 and #4

7.VIP

#1 题名或关键词：骨质疏松性椎体压缩性骨折 + 骨质疏松性骨折
#2 题名或关键词：经皮椎体成形术 + 椎体强化术 + 经皮椎体后凸成形术
#3 题名或关键词：再骨折 + 新发骨折 + 二次骨折 + 复发骨折
#4 题名或关键词：预测 + 评估 + 模型 + 筛查 + 因素
#5: #1 and #2 and #3 and #4
