# Supplementary material for: Risk Prediction Models for New Vertebral Fracture After Vertebral Augmentation in Elderly Patients with Osteoporotic Vertebral Compression Fractures: A Systematic Review
Source: Healthcare (Basel). 2026 Jul 17;14(14):2162. doi: 10.3390/healthcare14142162 (PMC13411060; doi:10.3390/healthcare14142162)
Supplement: Supplementary file 1 [file healthcare-14-02162-s001.zip › Supplementary Table S1.pdf]

Table S1.

Included study and publication year	Country	Study population	Study design	Sample size (n, modeling/validation n)	Follow-up duration	Type of New Vertebral Fracture	Incidence of new vertebral fracture (%)	Modeling method	Model presentation form
Zhou XL et al [11], 2025	China	Patients undergoing PVP	Single-center retrospective cohort study	300	12 months	Adjacent vertebral fracture	22.33	Logistic regression model	Nomogram
Shen Y et al [16], 2023	China	Patients undergoing PVP	Single-center retrospective cohort study	193/63	24 months	Adjacent vertebral fracture	21.76	Cox proportional hazards regression model	Nomogram
Li X et al [17], 2025	China	Patients undergoing PVP	Single-center retrospective cohort study	174/75	6 months	Not specified	15.26	Logistic regression model	Nomogram, risk scoring formula
Huang H et al [18], 2023	China	Patients undergoing PVP	Single-center retrospective cohort study	345	>24 months	Not specified	16.23	Logistic regression model	Risk scoring formula
Tan HT et al [19], 2023	China	Patients undergoing PVP	Single-center retrospective cohort study	295	24 months	Not specified	16.27	Logistic regression model	Nomogram
Li QJ et al [12], 2021	China	Patients undergoing PKP or PVP	Single-center retrospective	385	24 months	Adjacent vertebral fracture	10.60	Logistic regression model	Nomogram

Included study and publication year	Country	Study population	Study design	Sample size (n, modeling/validation *n*)	Follow-up duration	Type of New Vertebral Fracture	Incidence of new vertebral fracture (%)	Modeling method	Model presentation form
			cohort study						
Li WL et al [20], 2021	China	Patients undergoing PVP	Single-center retrospective cohort study	308/77	24–36 months	Not specified	15.06	Logistic regression model	Nomogram
Li KP et al [30], 2024	China	Patients undergoing PKP	Single-center retrospective cohort study	365	>12 months	Not specified	38.08	LASSO regression combined with Logistic regression model	Nomogram
Zhang N et al [21], 2024	China	Patients undergoing PKP or PVP	Single-center retrospective cohort study	150	12 months	Not specified	29.33	Logistic regression model	Risk scoring formula
He Y et al [22], 2025	China	Patients undergoing PKP or PVP	Single-center retrospective cohort study	268	12 months	Not specified	18.66	Logistic regression model	Risk scoring formula
Sun L et al [23], 2024	China	Patients undergoing PKP	Single-center retrospective cohort study	182	36 months	Not specified	19.78	Logistic regression model	Nomogram
Huang D et al	China	Patients undergoing PKP	Single-center retrospec	205	12 months	Not specified	15.12	Logistic regression model	Nomogram

Included study and publication year	Country	Study population	Study design	Sample size (n, modeling/validation *n*)	Follow-up duration	Type of New Vertebral Fracture	Incidence of new vertebral fracture (%)	Modeling method	Model presentation form
[24], 2025			tive cohort study						
Wang XL et al [25], 2023	China	Patients undergoing PKP	Single-center retrospective cohort study	155	12 months	Not specified	20.65	Logistic regression model	Nomogram, risk scoring formula
Zhou QF et al [13], 2023	China	Patients undergoing PVP	Single-center retrospective cohort study	179	12 months	Adjacent vertebral fracture	27.93	Logistic regression model	Nomogram
Gai JY et al [26], 2022	China	Patients undergoing PKP or PVP	Single-center retrospective cohort study	187	12 months	Not specified	27.27	Logistic regression model	Nomogram
Ma YM et al [27], 2023	China	Patients undergoing PKP	Single-center retrospective cohort study	439	>12 months	Not specified	12.98	Logistic regression model	Nomogram, web-based calculator
YANG et al [28], 2026	China	Patients undergoing PKP	Single-center retrospective cohort study	197	>12 months	Not specified	21.80	Logistic regression model	Nomogram
HAIBIER et al [14], 2025	China	Patients undergoing PVP	Single-center retrospective	288/124	6–12 months	Adjacent vertebral fracture	22.09	LASSO regression combine	Nomogram

Included study and publication year	Country	Study population	Study design	Sample size (n, modeling/validation)	Follow-up duration	Type of New Vertebral Fracture	Incidence of new vertebral fracture (%)	Modeling method	Model presentation form
			cohort study					d with Logistic regression model	
ZHANG et al [31], 2025	China	Patients undergoing PKP	Single-center retrospective cohort study	240/100	12 months	Not specified	13.24	LASSO regression combined with Logistic regression model	Nomogram
BAO et al [33], 2025	China	Patients undergoing PVP	Single-center retrospective cohort study	392/168	>6 months	Not specified	33.57	Random forest, XGBoost, support vector machine, Logistic regression, gradient boosting machine, multilayer perceptron	Machine learning model
MA et al [29], 2023	China	Patients undergoing PKP	Single-center retrospective cohort study	397/132	>24 months	Not specified	10.60	Logistic regression model	Nomogram, web-based calculator
ZHANG et al	China	Patients undergoing PKP	Single-center retrospec	225/94	24 months	Not specified	13.48	LASSO regression	Nomogram

Included study and publication year	Country	Study population	Study design	Sample size (n, modeling/validation)	Follow-up duration	Type of New Vertebral Fracture	Incidence of new vertebral fracture (%)	Modeling method	Model presentation form
[32], 2023			tive cohort study					combined with Logistic regression model	
MAO et al [15], 2023	China	Patients undergoing PKP	Single-center retrospective cohort study	436	24 months	Adjacent vertebral fracture	17.89	LASSO regression combined with Logistic regression model	Nomogram
BIAN et al [10], 2022	China	Patients undergoing PKP	Multicenter retrospective cohort study	413/149	>24 months	Not specified	24.20	Logistic regression model	Nomogram

Note: PVP, percutaneous vertebroplasty; PKP, percutaneous kyphoplasty. “Not specified” indicates that the study did not explicitly define the anatomical location of refracture, and the outcome included, but was not limited to, adjacent vertebral fractures.
