# Supplementary material for: Risk Prediction Models for New Vertebral Fracture After Vertebral Augmentation in Elderly Patients with Osteoporotic Vertebral Compression Fractures: A Systematic Review
Source: Healthcare (Basel). 2026 Jul 17;14(14):2162. doi: 10.3390/healthcare14142162 (PMC13411060; doi:10.3390/healthcare14142162)
Supplement: Supplementary file 1 [file healthcare-14-02162-s001.zip › Supplementary Table S2.pdf]

Table S2.

Predictor	Frequency (*n*)	Predictor	Frequency (*n*)
Demographic characteristics		Surgery-related factors	
Age	8 [10,11,15,17,21,24,25,32]	Bone cement leakage	14 [10–12,14–16,18,22–25,27,29,32]
Sex	5 [15,17,21,29,30]	Bone cement injection volume	9 [11–17,23,25]
Body mass index	3 [19,20,32]	Vertebral height recovery rate	5 [11,13,16,31,32]
Self-care ability	1 [30]	Bone cement distribution	4 [11–13,32]
Disease-related factors		Sagittal spinal imbalance	2 [22,25]
Bone mineral density	18 [10,11,13 – 16,18 – 28,32]	Preoperative anterior vertebral height	2 [31,32]
Number of fractured vertebrae	7 [15,18–21,23,33]	Interval from fracture to hospitalization/surgery	2 [14,27]
Cerebrovascular disease	2 [27,29]	Preoperative Cobb angle	1 [14]
History of previous fracture	2 [27,29]	Vertebral height difference before and after PKP	1 [23]
Intravertebral cleft sign	2 [14,16]	Change of kyphotic angle	1 [23]
Fracture at thoracolumbar junction	2 [10,15]	Initial fracture site	1 [13]
Diabetes mellitus	2 [15,33]	Treatment-related factors	
Hypertension	1 [33]	Anti-osteoporosis treatment status	9 [11–13,19–21,26,30,32]
Heart disease	2 [30,33]	Glucocorticoid use	4 [19,20,22,26]
History of scoliosis	1 [33]	Regular postoperative exercise	2 [21,26]
Gastrointestinal disease	1 [30]	Bisphosphonate medication	1 [18]
Chronic kidney disease	1 [33]	Other factors	
Mental disorder	1 [33]	Paraspinal muscle mass	2 [24,28]

Predictor	Frequency (*n*)	Predictor	Frequency (*n*)
Chronic obstructive pulmonary disease	1 [33]	History of alcohol consumption	1 [33]
Osteoarthritis	1 [33]		
Gout	1 [33]		
Tumor	1 [33]		
History of coronary stent implantation	1 [33]		
Trauma history	1 [33]		
Decreased serum 25-hydroxyvitamin D3	1 [21]		
Elevated serum β-crosslaps	1 [22]		
VAS pain score	1 [17]		

Note: VAS = Visual Analog Scale, a commonly used tool for pain assessment; Cobb angle is an indicator for evaluating the severity of scoliosis.
