# Supplementary material for: Risk Prediction Models for New Vertebral Fracture After Vertebral Augmentation in Elderly Patients with Osteoporotic Vertebral Compression Fractures: A Systematic Review
Source: Healthcare (Basel). 2026 Jul 17;14(14):2162. doi: 10.3390/healthcare14142162 (PMC13411060; doi:10.3390/healthcare14142162)
Supplement: Supplementary file 1 [file healthcare-14-02162-s001.zip › Supplementary Table S3.pdf]

Table S3.

Included study	Validation method	Model performance evaluation	Sensitivity	Specificity	Calibration	DCA
Zhou XL et al [11]	Internal validation	AUC=0.908	0.940	0.734	Calibration curve	No
Shen Y et al [16]	Internal validation, External validation	1-year postoperative AUC=0.820[b], 2-year AUC=0.772[b]; 1-year postoperative AUC=0.778[c], 2-year AUC=0.721[c]	Not reported	Not reported	H-L test + Calibration curve	Yes
Li X et al [17]	Internal validation	AUC=0.744[a]; AUC=0.809[b]	Not reported	Not reported	Calibration curve	No
Huang H et al [18]	Not reported	AUC=0.921	0.768	0.948	H-L test	No
Tan HT et al [19]	Internal validation	AUC=0.909	Not reported	Not reported	Calibration curve	Yes
Li QJ et al [12]	Internal validation	AUC=0.881	Not reported	Not reported	Calibration curve	Yes
Li WL et al [20]	Internal validation, External validation	AUC=0.796[b]; AUC=0.648[c]	Not reported	Not reported	Calibration curve	No
Li KP et al [30]	Internal validation	AUC=0.750	Not reported	Not reported	Calibration curve	Yes
Zhang N et al [21]	Not reported	AUC=0.869	0.846	0.583	H-L test	No
He Y et al [22]	Not reported	AUC=0.894	0.860	0.807	H-L test	No
Sun L et al [23]	Internal validation	C-index=0.818	Not reported	Not reported	H-L test + Calibration curve	Yes
Huang D et al [24]	Internal validation	AUC=0.976	Not reported	Not reported	Calibration curve	No
Wang XL et al [25]	Not reported	AUC=0.885	Not reported	Not reported	H-L test + Calibration curve	No

Included study	Validation method	Model performance evaluation	Sensitivity	Specificity	Calibration	DCA
Zhou QF et al [13]	Internal validation	C-index=0.952	Not reported	Not reported	Calibration curve	No
Gai JY et al [26]	Not reported	AUC=0.670	Not reported	Not reported	H-L test + Calibration curve	No
Ma YM et al [27]	Internal validation	AUC=0.927[a]; AUC=0.807[b]	Not reported	Not reported	Calibration curve	Yes
YANG et al [28]	Internal validation	AUC=0.861[a]; AUC=0.796[b]	Not reported	Not reported	H-L test + Calibration curve	Yes
HAIBIER et al [14]	Internal validation	AUC=0.839[a]; AUC=0.846[b]	0.898[a]; 0.906[b]	0.619[a]; 0.607[b]	H-L test + Calibration curve	Yes
ZHANG et al [31]	Internal validation	AUC=0.833[a]; AUC=0.771[b]	Not reported	Not reported	H-L test + Calibration curve	Yes
BAO et al [33]	Internal validation	RF AUC=0.990[a], RF AUC=0.880[b], LR AUC=0.870[b], XGBoost AUC=0.870[b], GBM AUC=0.880[b], MLP AUC=0.880[b], SVM AUC=0.860[b]	RF 0.980[a], 0.770[b]; LR 0.880[b]; XGBoost 0.840[b]; GBM 0.880[b]; MLP 0.790[b]; SVM 0.750[b]	RF 0.940[a], 0.870[b]; LR 0.750[b]; XGBoost 0.770[b]; GBM 0.780[b]; MLP 0.830[b]; SVM 0.790[b]	Calibration curve + Brier score: 0.12	Yes
MA et al [29]	Internal validation	AUC=0.795[a]; AUC=0.861[b]	Not reported	Not reported	Calibration curve	Yes
ZHANG et al [32]	Internal validation	AUC=0.881[a]; AUC=0.929[b]	Not reported	Not reported	Calibration curve	Yes
MAO et al [15]	Internal validation	AUC=0.886[a]; AUC=0.833[b]	Not reported	Not reported	Calibration curve	Yes
BIAN et al [10]	Internal validation, External validation	AUC=0.850[a]; AUC=0.880[b]; AUC=0.870[c]	Not reported	Not reported	Calibration curve	Yes

Notes: AUC=area under the curve; C-index=concordance index; H-L test=Hosmer-Lemeshow test; RF=random forest; LR=logistic regression; XGBoost=extreme gradient boosting; GBM=gradient boosting machine; MLP=multilayer perceptron; SVM=support vector machine;

PKP=percutaneous kyphoplasty; VAS=visual analogue scale; a=training set; b=test set; c=external validation set; BMD=Bone mineral density.
